# Prevalence of endometrial polyps coexisting with uterine fibroids and associated factors

**DOI:** 10.4274/tjod.36043

**Published:** 2016-03-10

**Authors:** Tuğba Kınay, Zehra Öztürk Başarır, Serap Fırtına Tuncer, Funda Akpınar, Fulya Kayıkçıoğlu, Sevgi Koç

**Affiliations:** 1 Etlik Zübeyde Hanım Women’s Health Training and Research Hospital, Clinic of Obstetrics and Gynecology, Ankara, Turkey; 2 Zekai Tahir Burak Women’s Health Training and Research Hospital, Clinic of Obstetrics and Gynecology, Ankara, Turkey

**Keywords:** Cervical polyps, condom, endometrial hyperplasia, endometrial polyps, uterine fibroids

## Abstract

**Objective:**

The aim of the study was to investigate the prevalence of endometrial polyps in patients with uterine fibroids and associated factors of coexistence of these two pathologies.

**Materials and Methods:**

The medical records of 772 patients who underwent hysterectomy because of uterine fibroids were retrospectively reviewed. Patients were divided into two groups according to the presence of endometrial polyps in the histopathologic examination. Demographic, clinical and histopathologic findings of the patients with and without endometrial polyps were compared. Student’s t-test, Mann-Whitney U test, Pearson’s Chi-square test, and logistic regression analysis were used for statistical analysis.

**Results:**

The prevalence of the endometrial polyps in uterine fibroid cases was found 20.1% (n=155). Age ≥45 years (odds ratio [OR] 1.61; 95% confidence interval [CI]: [1.06-2.44]; p=0.014), presence of hypertension (23.9% vs. 17.5%; p=0.047), endometrial hyperplasia (OR 4.00; 95% CI: [1.92-8.33]; p<0.001) and cervical polyps (OR 3.13; 95% CI: [1.69-5.88]; p<0.001) were significantly associated with the coexistence of endometrial polyps and uterine fibroids. Endometrial polyps were more common in patients with ≥2 fibroids (p=0.023) and largest fibroid <8 cm (p=0.009). A negative correlation was found between condom use and endometrial polyps (8.1% vs. 3.9%; p=0.044).

**Conclusions:**

The prevalence of the endometrial polyps coexisting with uterine fibroids was 20.1%. Age, hypertension, endometrial hyperplasia, cervical polyps, and number of fibroids were positively correlated; condom use and size of largest fibroid were negatively correlated with the coexistence of these two pathologies.

## PRECIS:

We investigated the prevalence of the endometrial polyps in patients with uterine fibroids and associated factors with the coexistence of these two pathologies.

## INTRODUCTION

Endometrial polyps and uterine fibroids are common causes of abnormal uterine bleeding and may coexist. Overgrowth of endometrial glands and stroma leads to endometrial polyps. The prevalence of endometrial polyps is 10-40% in women with abnormal uterine bleeding, and increases with age^(1,2)^.Transvaginal sonography, saline infusion sonohysterography, and hysteroscopy are the diagnostic tools for endometrial polyps^(3,4)^. Malignant tissue changes occur in 3.1% of endometrial polyps^(1)^. Therefore, histopathologic examination of polyps is necessary to exclude malignancy^(5,6)^.

Uterine fibroids are the most common benign tumors in women. Abnormal uterine bleeding occurs in 30% of women with fibroids^(7)^. Uterine fibroids are the leading causes of hysterectomy, and hysterectomy is the most effective treatment method for symptomatic fibroids in perimenopausal women^(8,9)^. However, alternative treatments such as progestogens, levonorgestrel-releasing intrauterine system, tranexamic acid, nonsteroidal anti-inflammatory drugs, gonadotropin-releasing hormone analogs, myomectomy, and uterine artery embolization are also available in the management of uterine fibroids^(9)^.

As far as we know, no studies have investigated the coexistence of endometrial polyps and uterine fibroids in the literature. The aim of the retrospective study was to identify the prevalence of the endometrial polyps coexisting with uterine fibroids and associated factors of the coexistence of the two pathologies. Knowledge of the coexistence of these pathologies may help to choose the appropriate therapeutic management. Medical management, myomectomy, or uterine artery embolization are not appropriate treatment options in the presence of malignant endometrial polyps, and hysteroscopic resection alone of endometrial polyps may not be sufficient to relieve abnormal uterine bleeding symptoms in patients with multiple and large uterine fibroids.

## MATERIALS AND METHODS

Women who underwent hysterectomy due to uterine fibroid at a training and research hospital in Turkey, between January 2009 and December 2013, were included the retrospective study. Data were collected from medical records. The study was approved by the institutional review board and written informed consent was obtained from all participants. Exclusion criteria were hysterectomy because of gynecologic malignancy, benign ovarian neoplasm, and uterine prolapse. Patients who underwent myomectomy were also excluded.

Uterine fibroids were diagnosed using preoperative transvaginal ultrasonography, and confirmed through histopathologic examination of hysterectomy specimens, and endometrial polyps were diagnosed through histopathologic examination of the preoperative endometrial biopsy or hysterectomy specimens. Premenopausal patients with a history of abnormal uterine bleeding, and postmenopausal patients with bleeding symptoms and/or endometrial thickness ≥5 mm in ultrasonographic examination underwent preoperative endometrial biopsy. The women were divided into two groups: the study group consisted of women with endometrial polyps and uterine fibroids; and the control group comprised women with uterine fibroids alone. Women’s age, body mass index, gravidity, parity, menopausal status, contraception methods, preoperative hemoglobin levels, history of cesarean section, smoking, hypertension, diabetes mellitus, and abnormal uterine bleeding were compared between the two groups.

Heavy menstrual bleeding, intermenstrual bleeding, and postmenopausal bleeding were defined as abnormal uterine bleeding. Regular or irregular bleeding >7 days and/or >80 mL were considered heavy menstrual bleeding. Spotting or more bleeding among regular menses was considered intermenstrual bleeding. The number of uterine fibroids, size and location of the largest fibroid, and other accompanying gynecologic proliferative pathologies (adenomyosis, endometrial hyperplasia, cervical polyps, and endometriosis) were recorded from the womens’ histopathology reports.

Statistical analysis was performed usig Statistical Package for Social Science version 15.0 (SPSS Inc., Chicago, IL, USA) software. The normality for continuous variables was checked by using Kolmogorov-Smirnov test. Descriptive statistics are presented as mean ± standard deviation or median (minimum-maximum). Case numbers and percentages are given for categorical variables. Student’s t-test or Mann-Whitney U test were used for the comparison of continuous data. Pearson’s Chi-square test was used to examine the differences between groups for categorical variables. The odds ratio (OR) of endometrial polyps coexisting with uterine fibroids and 95% confidence interval (CI) were calculated using logistic regression analysis. Statistical significance was accepted as p<0.05.

## RESULTS

A total of 772 women were analyzed: 155 women with endometrial polyps and uterine fibroids, and 617 women with uterine fibroids alone. The prevalence of endometrial polyps in women with uterine fibroid was 20.1%. The demographic and clinical characteristics of women and contraception methods used are shown in Table 1. Increased age was found as a risk factor for endometrial polyps coexisting with uterine fibroids. The endometrial polyp rate was significantly higher in women aged ≥45 years (OR 1.61; 95% CI: [1.06-2.44]). Hypertension was more common (23.9% vs. 17.5%; p=0.047) in women with endometrial polyps and uterine fibroids. When contraceptive methods were investigated, a protective effect of condom use was found. Condom use was more common in women without endometrial polyps (8.1% vs. 3.9%; p=0.044).

The median uterine sizes of both groups were equivalent to 12 weeks pregnancy in the genital examination, ranging from 6-22 weeks in patients with endometrial polyps, and 6-24 weeks in patients without endometrial polyps; p=0.368). As shown in Table 2, no statistical differences were found between the symptoms of women with and without endometrial polyps. Abnormal uterine bleeding rates (56.1% vs. 57.1%; p=0.453), and the mean preoperative hemoglobin levels (11.2±2.4 g/dL vs. 11.4±4.5 g/dL; p=0.874) were similar in both groups.

Table 3 shows the association between the histopathologic findings and the coexistence of endometrial polyps and uterine fibroids. The endometrial polyp rate was higher in women with ≥2 fibroids (OR 1.51; 95% CI: [1.02-2.24]) and with a largest fibroid <8 cm (OR 1.67; 95% CI: [1.10-2.50]). Presence of endometrial hyperplasia (OR 4.00; 95% CI: [1.92-8.33]) and cervical polyps (OR 3.13; 95% CI: [1.69-5.88]) were found significantly associated with the coexistence of endometrial polyps and uterine fibroids. Women with endometrial polyps had higher endometrial hyperplasia rates (9.7% vs. 2.6%; p<0.001) and cervical polyp rates (12.3% vs. 4.2%; p<0.001) than women with uterine fibroids alone. There was no relationship between adenomyosis, endometriosis, and endometrial polyps coexisting with uterine fibroids (p>0.05).

## DISCUSSION

Endometrial polyps are one of the common causes of abnormal uterine bleeding in premenopausal and postmenopausal women, and its incidence increases with age^(10)^. The pathogenesis of this pathology is not known exactly. Presence of estrogen and progesterone receptors in polyp specimens suggests that the increased endogenous and exogenous estrogen level plays a role in the endometrial polyp growth^(11)^. In the literature, association between endometrial polyps and tamoxifen use in breast cancer, postmenopausal hormone therapy, and obesity has been shown^(12,13,14)^. Unopposed, high estrogen levels increase the insulin-like growth factor (IGF)-1 level, and the number of IGF-1 receptors within the endometrial tissue and causes endometrial polyp growth^(15^). Studies reported that hypertension and hyperglycemia also induce endometrial polyp growth by locally modifying the expression of IGF^(2,16)^. We investigated the risk factors for the coexistence of endometrial polyps and uterine fibroids and found that the mean age and hypertension rates were significantly higher in women with endometrial polyps coexisting with uterine fibroids. However, there was no relationship between diabetes mellitus, obesity, and coexistence of these two pathologies.

The present study showed the protective effect of condom use against endometrial polyp growth. The rate of condom use was lower in women with endometrial polyps. Cicinelli et al.^(17)^ reported that the presence of endometrial micropolyps was significantly associated with chronic endometritis. Expression of cyclooxygenase-2 and matrix metalloproteinase-2 in immunohistochemical analyses of endometrial polyps has also been shown in some studies^(18,19)^. These findings suggest that the endometrial polyps may have an inflammatory etiopathogenesis. An inflammatory response to sexually transmitted microorganisms or seminal antigens may play a role in the etiology of endometrial polyps. Korucuoglu et al.^(20)^ reported that human papillomavirus (HPV) infection might cause endometrial polyp growth. Condom use inhibits endometrial polyp growth by preventing the inflammatory response to these antigens.

In the present study, endometrial polyps were more common in women with ≥2 fibroids and largest fibroid size <8 cm. The endometrial cavity should be examined using transvaginal ultrasonography, endometrial biopsy, and saline infusion sonohysterography and/or hysteroscopy in the presence of suspected premalignant and malignant endometrial polyp, before choosing the treatment method. Savelli et al.^(21)^ reported that hyperplastic changes were more common in endometrial polyps. In another study, 248 women with abnormal uterine bleeding were investigated, and hyperplasia was found more common in women with endometrial polyps^(22)^. Similarly, we found an association between endometrial hyperplasia,cervical polyps, and the coexistence of endometrial polyps and uterine fibroids^(21,22,23)^.

Indraccolo and Barbieri reported that adenomyosis was associated with endometrial polyps^(24)^. Their study had limitations due to the method used to obtain specimens. Adenomyosis was diagnosed in histopathologic examinations of specimens obtained in deep biopsies of the endometrium, reaching the deeper myometrial layer during hysteroscopic resection of endometrial polyps. However, in our study, specimens obtained with hysterectomy were examined and we found no association between endometrial polyps and adenomyosis. Shen et al.^(25)^ noted that the endometrial polyp rate was higher in infertile patients with endometriosis. The authors suggested that patients with endometriosis should be evaluated using hysteroscopy. Our study population included fertile and infertile women, and no association was found between endometriosis and endometrial polyps. Even though approximately 95% of endometrial polyps are benign,resection of endometrial polyps is recommended to rule out malignancy^(1,5)^. The risk of malignancy and the presence of abnormal uterine bleeding increases in the postmenopausal period^(1,26,27,28)^. Histopathologic examinations of endometrial polyps provide the selection of optimal treatment methods in patients with both endometrial polyps and uterine fibroids. Medical management, myomectomy or uterine artery embolization are not appropriate treatment methods in the presence of malignant endometrial polyps.

Uterine fibroids and endometrial polyps are common causes of abnormal uterine bleeding in the reproductive period^(29)^. Hysterectomy is the definitive therapy for uterine fibroids, whereas hysteroscopic resection is the optimal treatment method for endometrial polyps to reduce the cost and morbidity associated with surgery^(1,9)^. In the study population, abnormal uterine bleeding rates were similar in women with endometrial polyps and uterine fibroids, and with uterine fibroids alone, and the median uterine size was the equivalent of 12 weeks pregnancy in both groups. Therefore, it was considered that just hysteroscopic polypectomy may not be enough to relieve the abnormal uterine bleeding symptoms and treatment of uterine fibroids in women with both endometrial polyps and fibroids with uteri greater than 12 weeks-pregnancy in size. Nevertheless, further randomized controlled trials are needed to confirm this hypothesis. The present study was not designed to investigate the impact of fibroid size, number, and location in the success of treatment in patients with endometrial polyps and uterine fibroids.

As far as we know, ours is the first study to investigate the coexistence of endometrial polyps and uterine fibroids, and is one of the largest studies on uterine fibroids. However, the major limitation is the retrospective design of the study. Data were obtained retrospectively and control and study groups were nonhomogeneous. Therefore, it is not known whether the defined risk factors were independent risk factors for the coexistence of endometrial polyps and uterine fibroids.

## CONCLUSION

The present study showed that age, number of fibroids, size of the largest fibroid, presence of hypertension, endometrial hyperplasia, and cervical polyps were associated factors with the coexistence of endometrial polyps and uterine fibroids. Condom use has a protective effect against endometrial polyp growth in these patients.

## Figures and Tables

**Table 1:**
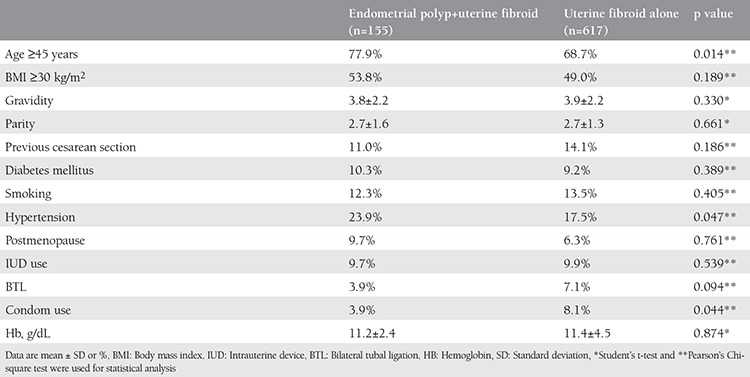
Demographic, clinical characteristics, and contraception methods of women with endometrial polyps and uterine fibroids, and with uterine fibroids alone

**Table 2:**
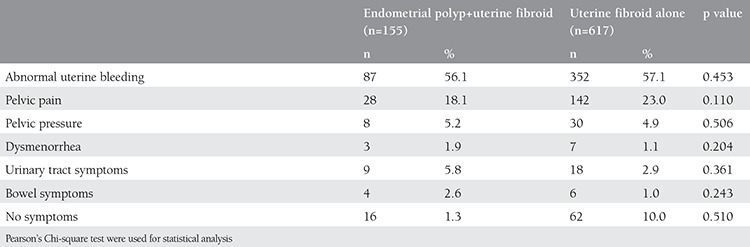
Symptoms of women with endometrial polyp and uterine fibroid and with uterine fibroid alone

**Table 3:**
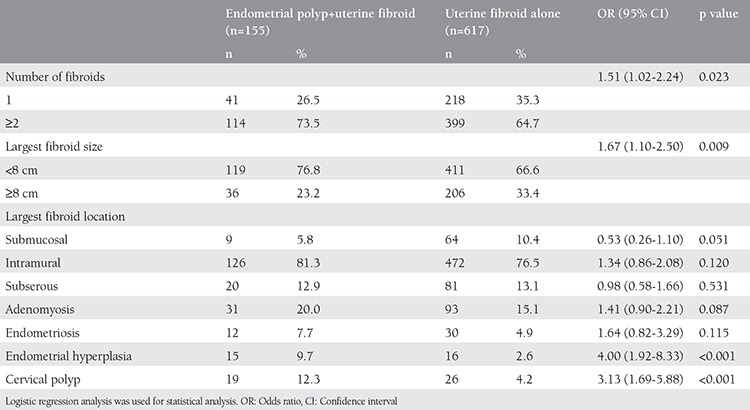
Histopathologic findings of women with endometrial polyp and uterine fibroid and with uterine fibroid alone
